# Prevalence and predictors of 6-month exclusive breastfeeding among Canadian women: a national survey

**DOI:** 10.1186/1471-2431-10-20

**Published:** 2010-04-08

**Authors:** Ban Al-Sahab, Andrea Lanes, Mark Feldman, Hala Tamim

**Affiliations:** 1 Kinesiology & Health Science, York University, Toronto, Ontario, Canada; 2 Community Paediatrics, University of Toronto & Department of Paediatrics, St Joseph's Health Centre, Toronto, Ontario, Canada

## Abstract

**Background:**

In spite of the evidence supporting the importance of breastfeeding during the first year of life, data on breastfeeding practices remain limited in Canada. The study aimed to examine the prevalence and predictors of 6-month exclusive breastfeeding among Canadian women.

**Methods:**

The analysis was based on the Maternity Experience Survey targeting women aged ≥ 15 years who had singleton live births between February 2006 - May 2006 in the Canadian provinces and November 2005 - February 2006 in the territories. The main outcome was exclusive breastfeeding based on the World Health Organization definition. Socioeconomic, demographic, maternal, pregnancy and delivery related variables were considered for a multivariate logistic regression using stepwise modeling. Bootstrapping was performed to account for the complex sampling design.

**Results:**

The sample size in this study was 5,615 weighted to represent 66,810 Canadian women. While ever breastfeeding was 90.3%, the 6-month exclusive breastfeeding rate was 13.8%. Based on the regression model, having higher years of education, residing in the Northern territories and Western provinces, living with a partner, having had previous pregnancies, having lower pre-pregnancy body mass index and giving birth at older age were associated with increased likelihood of 6-month exclusive breastfeeding. Moreover, smoking during pregnancy, Caesarean birth, infant's admission to the intensive care unit and maternal employment status before 6 months of infant's age were negatively associated with exclusive breastfeeding. Mothers choosing to deliver at home were more likely to remain exclusively breastfeeding for 6 months (Odds Ratio: 5.29, 95% Confidence Interval: 2.95-9.46).

**Conclusions:**

The 6-month exclusive breastfeeding rate is low in Canada. The study results constitute the basis for designing interventions that aim to bridge the gap between the current practices of breastfeeding and the World Health Organization recommendation.

## Background

Epidemiological research provides compelling evidence for the effect of human milk in decreasing the risk of infant mortality and morbidity from acute and chronic diseases [1-3]. The World Health Organization (WHO) advocates for breastfeeding as the best source of food for optimal infant growth and development. They recommend that infants should be exclusively breastfed, receiving no other foods or liquids besides breast milk, until 6 months of age [4,5]. Although there is a debate that infants exclusively breastfed for 6 months are subject to energy and micronutrients deficiency (particularly iron and zinc) [6,7], the Canadian Paediatric Society, Health Canada and Public Health Agency of Canada have adopted the WHO 6-month exclusive breastfeeding recommendation [8].

The prevalence of breastfeeding among women has been shown to vary substantially across the provinces of Canada. The initiation of breastfeeding ranges from 91.1% in Ontario [9] to 85.6% in Alberta [10] and 72% in Quebec [11]. Breastfeeding continuation up to 6 months was reported to be 22.8% in Southwestern Ontario [12], 37.2% in Alberta [10] and 32% in Montérégie, Quebec [13]. The prevalence of exclusive breastfeeding at 6 month, however, is much lower. Millar & Maclean (2005) reported that only 17% of women in Canada conform with the 6-month exclusive breastfeeding recommendation of the WHO. Based on the Canadian studies, exclusive breastfeeding was significantly more common among urban residents [14], women with high education [11,14] and older mothers [11,14]. Risk factors for early breastfeeding termination were also found to be associated with early hospital discharge, minimal breastfeeding support and receiving advice on formula feeding [9].

Acquiring information on the predictors of breastfeeding may better equip policy makers and public health practitioners in designing programs for at-risk groups and may help to bring the entire population closer to the infant feeding practices recommended by Health Canada and the WHO. In spite of the evidence supporting the importance of breastfeeding during the first year of life and the variety of health outcomes that are related to breastfeeding, data on breastfeeding practices remain limited in Canada. Canadian studies are mostly representative of specific regions/provinces [10,12,13,15-17] and specific populations such as teenagers [18], low income mothers [19], female physicians [20] and primipara mothers [21]. To our knowledge, only one nationwide study, using data from 2003, assessed the prevalence and predictors of breastfeeding across the Canadian provinces [14]. The study, however, excluded mothers in the northern territories. It also assessed breastfeeding status within the previous 5 years thereby increasing the chance of recall bias. The study, as well, investigated limited demographic and socio-economic predictors. The present study, however, used data from a recent specialized survey on pre and post delivery experiences among mothers residing in both the Canadian provinces and territories. It aimed to examine the prevalence of exclusive breastfeeding at 6 months and the potential socio-economic, demographic, maternal, pregnancy and delivery related predictors.

## Methods

The analysis of this study was based on the Maternity Experience Survey (MES) that was sponsored by Public Health Agency of Canada and conducted by Statistics Canada in 2006. The MES study is the first nationwide survey that assessed pregnancy, delivery and postnatal experiences of mothers and their children. The study sample was selected from the Canadian Census of Population to include women aged ≥ 15 years who had singleton live births between February 15, 2006 and May, 2006 in the provinces of Canada and November 1, 2005 and February 1, 2006 in the territories of Canada. A total of 8,542 Canadian women were selected, out of which 6,421 (75.2%) responded to the survey. The data was collected through telephone interviews using a computer-assisted telephone interview application. Interviews were conducted between the 5^th ^and 14^th ^month after delivery and lasted on average 45 minutes. The majority (96.9%) of the interviews, however, were performed between the 5^th ^and 9^th ^month postpartum. The MES has been previously described in other references [22].

The present study considered the 5615 MES mothers (87.4%) who had babies aged ≥ 6 month at the time of interview. Mothers were weighted to represent 66,810 Canadian women. The main outcome of the study was exclusive breastfeeding based on the WHO definition as the intake of breast milk only without any other drink or food for the first 6 months of infant's age [5]. This outcome was dichotomous (<6 months, ≥ 6 months) and was calculated using information about breastfeeding termination and timing of introduction of liquids, semi-solid and solid foods. Other breastfeeding variables that were considered were, ever breastfeeding assessed by the question "did you breastfeed or try to breastfeed even if only for a short time?" and breastfeeding intention measured by the question "prior to giving birth, did you intend to feed by formula alone, breastfeeding alone or a combination of both?"

A wide range of variables were investigated as potential predictors of exclusive breastfeeding. Socio-economic status, such as maternal years of education, total household income and place of residence, and demographic factors, consisting of immigration status and province of residence, were considered. Information about maternal characteristics including marital status, age at first pregnancy, number of previous pregnancies, age at selected birth, pre-pregnancy maternal body mass index (BMI) and mother's perceived health were also assessed. Furthermore, pregnancy related factors composed of: self reported weight gain during pregnancy, ever taking alcohol during pregnancy, ever smoking during the third trimester of pregnancy, support during pregnancy, mother's reaction to pregnancy, mother's stress level before and during pregnancy, health problems during pregnancy, attendance of prenatal classes, number of prenatal care visits and type of prenatal care provider were explored as well. Finally, delivery related factors (type of delivery, type of birth setting, birth weight, gestational age and baby's admission to neonatal intensive care unit) and postpartum variables (hospitalization of baby, support after birth, work status after birth and postpartum depression) were examined. All the variables, except for mother's stress level and postpartum depression, were directly self-reported by the mother. The mother's stress level was measured through a set of 13 questions that examined the mother's experience of stressful events in the past 12 months before the birth of her selected child. The questions were adapted by Pregnancy Risk Assessment Monitoring System (PRAMS) from Newton and Hunt's Life Events Inventory [23]. The answers for these questions were categorised as "Yes" or "No". Consequently, the sum of the "Yes" responses was calculated for each mother to represent her stress level [24]. Postpartum depression, on the other hand, was assessed using the Edinburgh Postpartum Depression Scale [25]. The scale consists of 10 items with four response categories scored from 0 to 3, whereby the highest values represent depressed moods. The sum of scores represents the mother's level of postpartum depression [24].

The prevalence of exclusive breastfeeding was estimated through population weights and examined across all the Canadian provinces and territories. At the bivariate level, differences in the proportion of exclusive breastfeeding were assessed among the different levels of each predictor using normalized weights. Chi square tests and odds ratios (OR) using 95% confidence intervals (95% CI) were performed for categorical variables. Differences in means and 95% confidence interval estimations were employed for continuous variables. All the independent variables were considered for a multivariate logistic regression analysis using stepwise modeling. Adjusted OR and 95% CI were reported for the final model. To account for the complex sampling design, bootstrapping was performed to calculate the 95% CI estimates. Population weights, normalized weights and bootstrap weights were all created by Statistics Canada and provided with the MES data file. All analyses, in exception to bootstrapping, were conducted using the Statistical Package for Social Sciences (SPSS, version 17.0). Bootstrapping was performed using the Statistical Analysis Software (SAS, version 9.2). Statistical significance for all analyses was set at alpha <0.05 for a two tailed tests.

## Results

**Figure 1:**
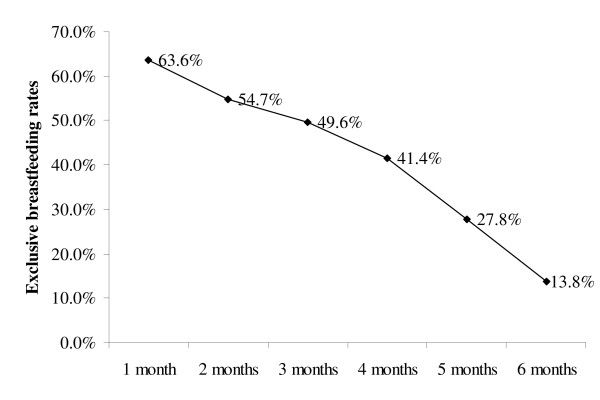
**Exclusive breastfeeding rates during the first 6 months of life across the Canadian provinces and territories (2005/06)**.

**Figure 2:**
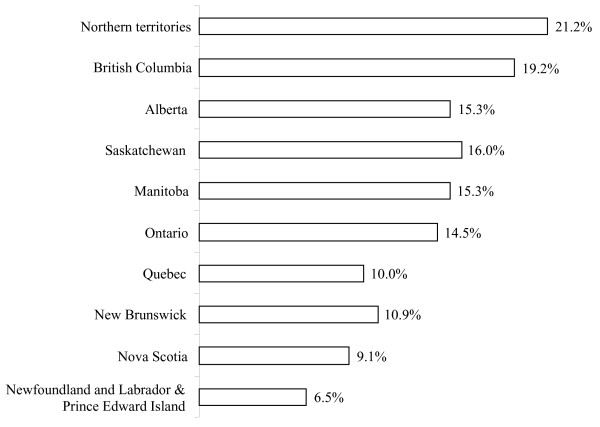
**Distribution of 6-month exclusive breastfeeding rates across the Canadian provinces and territories (2005/06)**.  Note: P-value < 0.001

Table 1 presents the estimated population and distribution of breastfeeding related outcomes. During pregnancy, around 90% of the women intended to breastfeed their child. Exclusive breastfeeding rates from 1 to 6 months are illustrated in Figure 1. At 1 month, the exclusive breastfeeding rates were 63.6% (95% CI: 62.3%-64.9%). By 3 months, half of the Canadian women were exclusively breastfeeding (50.4%, 95% CI: 48.2%-50.9%). The 6-month exclusive breastfeeding rate was 13.8% (95% CI: 12.9-14.8) while more than half of the women remained breastfeeding at 6 months of infant's age. Figure 2 compares the breastfeeding rates across the Canadian provinces and territories (P-value < 0.001). The Northern Territories and British Columbia demonstrated the highest prevalence of exclusive breastfeeding at 6 months (21.2% and 19.2%, respectively). The rate in Newfoundland and Labrador and Prince Edward Islands, on the other hand, was the lowest at 6.5%.

**Table 1:** Estimated frequency distribution of breastfeeding related variables

	N*	% (95% CI)†
Intention of breastfeeding before child birth		
Formula feeding alone	6,610	9.9 (9.2-10.7)
Breastfeeding alone	49,850	75.0 (73.8-76.1)
Combination of formula & breastfeeding	10,027	15.1 (14.1-16.1)
Ever breastfeeding	60,309	90.3 (89.6-91.1)
Liquids were first introduced at ≥ 6 months	17,182	25.8 (24.6-27.0)
Solids were first introduced at ≥ 6 months	21,306	31.9 (30.6-33.2)
Breastfeeding termination at ≥ 6 months	35,946	53.9 (52.6-55.2)
Exclusive breastfeeding for ≥ 6 months	9,217	13.8 (12.9-14.8)

* Sample size is estimated using population weights

† 95% CI were calculated using bootstrapping technique

Unadjusted associations between exclusive breastfeeding and potential predictors are shown in Table 2. Out of the 30 variables that were considered for stepwise logistic regression, 12 variables were retained in the final model (Table 3). Years of education was the only significant socioeconomic variable (OR: 1.08, 95% CI: 1.05-1.12). Out of the demographic variables tested, province of residence remained in the model. As compared to Eastern Atlantic provinces, the residents of Northern Territories and British Columbia were 3.01 (95% CI: 2.21-4.12) and 1.94 (95% CI: 1.42-2.64), respectively, more likely to exclusively breastfeed for the first 6 months of the infant's life. Mothers with partners, mothers with lower BMI before pregnancy, mothers who had more pregnancies and mothers who had their first pregnancy at an older age also had an increased likelihood of breastfeeding exclusively for 6 months. Furthermore, smoking during pregnancy was negatively associated with exclusive breastfeeding (OR: 2.11, 95% CI: 1.36-3.27). Women giving birth at home were 5 times more likely to exclusively breastfeed than those who gave birth at hospitals or clinics. Vaginal delivery was also found to increase the exclusive breastfeeding rates at 6 months by 25% as compared to Caesarean delivery (OR: 1.25, 95% CI: 1.01-1.53). Finally, mothers who had their babies admitted to neonatal intensive care unit after birth and mothers who returned to work within the first 6 postpartum months were less likely to achieve 6-month exclusivity of breastfeeding.

**Table 2:** Unadjusted associations between 6-month exclusive breastfeeding and potential predictors

	Sample size N*	Exclusive breast-feeding N* (%)	Unadjusted odds ratio OR (95% CI)†
Household income (Canadian dollar)			
<$30,000	908	98 (10.8)	1
$30,000 to less than $60,000	1,640	194 (11.8)	1.11 (0.84-1.47)
$60,000 to less than $100,000	1,657	231 (13.9)	**1.35 (1.02-1.77)**
≥ $100,000	1,052	213 (20.2)	**2.11 (1.60-2.79)**
Place of residence			
Rural area	973	129 (13.3)	1
Urban, population ≤ 499,999	1,976	247 (12.5)	0.93 (0.75-1.17)
Urban, population ≥ 500,000	2,436	363 (14.9)	1.15 (0.92-1.43)
Immigrant mother			
No	4,334	559 (12.9)	1
Yes	1,239	207 (16.7)	**1.36 (1.12-1.64)**
Marital status			
No partner	473	27 (5.7)	1
Have a partner	5,105	743 (14.6)	**2.79 (1.86-4.18)**
Moms perceived health			
Excellent/very good	4,022	596 (14.8)	**2.11 (1.30-3.43)**
Good	1,274	154 (12.1)	1.67 (0.99-2.80)
Poor/Fair	304	23 (7.6)	1
Reaction when discovered pregnancy			
Very happy/happy	5,184	722 (13.9)	1
Indifferent	238	27 (11.3)	0.78 (0.50-1.21)
Very unhappy/Unhappy	164	21 (12.8)	0.89 (0.54-1.48)
Smoking during pregnancy			
No	4,982	740 (14.9)	**3.15 (2.12-4.68)**
Yes	607	32 (5.3)	1
Alcohol drinking during pregnancy			
No	4,982	677 (13.6)	0.82 (0.64-1.06)
Yes	586	94 (16.0)	1
Health problems during pregnancy			
No	4,232	610 (14.4)	**1.23 (1.01-1.50)**
Yes	1,365	164 (12.0)	1
Support during pregnancy			
None/Little of time	289	37 (12.8)	0.92 (0.61-1.40)
Some of the time	448	69 (15.4)	1.14 (0.86-1.52)
Most/All of time	4,846	666 (13.7)	1
Attended prenatal classes			
No	3,770	520 (13.8)	1
Yes	1,830	253 (13.8)	1.00 (0.85-1.18)
Prenatal care provider			
Non-physician	397	102 (25.7)	**2.34 (1.81-3.02)**
Physician	5,175	668 (12.9)	1
Type of setting of baby's birth			
Hospital or clinic	5,485	728 (13.3)	1
Birthing centre	43	7 (16.3)	1.22 (0.44-3.39)
Private home	71	37 (52.1)	**7.13 (4.24-11.98)**
Type of delivery			
Vaginal	4,146	605 (14.6)	**1.31 (1.08-1.58)**
Caesarean	1,456	168 (11.5)	1
Baby's admission to NICU			
No	4,875	711 (14.6)	**1.83 (1.38-2.44)**
Yes	722	62 (8.6)	1
Baby's hospitalization after birth			
No	5,167	727 (14.1)	**1.46 (1.03-2.07)**
Yes	432	44 (10.2)	1
Mother's work status <6 months of delivery			
No	5,084	715 (14.1)	1.30 (0.97-1.74)
Yes	491	55 (11.2%)	1
Support after birth			
None/Little of time	322	61 (18.9)	**1.50 (1.07-2.09)**
Some of the time	583	80 (13.7)	1.02 (0.77-1.33)
Most/All of time	4,687	633 (13.5)	1
Province‡			
Eastern- Atlantic	323	28 (8.7)	1
Eastern- Central	3,523	448 (12.7)	**1.52 (1.21-1.91)**
Western- Prairies	1,056	163 (15.4)	**1.89 (1.46-2.45)**
Western- British Columbia	668	128 (19.2)	**2.46 (1.83-3.30)**
Northern territories	33	7 (21.2)	**2.69 (2.06-3.53)**
		**Unadjusted Mean difference**§ **(95% CI)**†	
Mother's education level (years)	5,538	**1.20 (0.97-0.43)**	
Age at first pregnancy (years)	5,527	**1.88 (1.47-2.29)**	
Number of past pregnancies	5,581	**0.16 (0.04-0.28)**	
Mother's age at selected birth (years)	5,581	**2.04 (1.67-2.41)**	
Weight gained during pregnancy (Kg)	5,535	-0.41 (-0.94-0.12)	
BMI before pregnancy (Kg/m^2^)	5,508	**-0.90 (-1.31--0.48)**	
Infant's birth weight (grams)	5,590	**44.39 (0.05-0.30)**	
Gestational age (weeks)	5,419	**0.17 (0.95-87.84)**	
Number of stressful events	5,556	**-0.23 (-0.33--0.12)**	
Number of prenatal visits	5,364	-0.07 (-0.41-0.27)	
Edinburgh Postnatal Depression Scale	5,529	-0.28 (-0.63-0.08)	

* Sample size is estimated using normalized weights

† 95% CI were calculated using bootstrapping technique

‡ Eastern Atlantic: Newfoundland & Labrador, Nova Scotia, Prince Edward Island & New Brunswick; Eastern Central: Quebec & Ontario; Western Prairies: Manitoba, Saskatchewan, & Alberta; Western British Columbia: British Columbia; and Northern Territories: Yukon Territory, Nunavut & Northwest Territories.

§Represents the difference between the mean of exclusive breastfeeders and non- exclusive breastfeeders (Mean_exclusive breastfeeders _- Mean_non-exclusive breastfeeders)_.

**Table 3:** Stepwise logistic regression model for the potential predictors of 6-month exclusive breastfeeding

	Adjusted odds ratio **OR (95% CI)**†
Marital status	
No partner	1
Have a partner	**1.61 (1.03-2.52)**
Moms perceived health	
Excellent/very good	1.59 (0.92-2.75)
Good	1.45 (0.82-2.57)
Poor/Fair	1
Smoking during pregnancy	
No	**2.11 (1.36-3.27)**
Yes	1
Type of setting of baby's birth	
Hospital or clinic	1
Birthing centre	1.20 (0.42-3.39)
Private home	**5.29 (2.95-9.46)**
Type of delivery	
Vaginal	**1.25 (1.01-1.53)**
Caesarean	1
Baby's admission to NICU	
No	**1.51 (1.12-2.03)**
Yes	1
Mother's employment status <6 months of delivery	
No	**1.55 (1.14-2.10)**
Yes	1
Province‡	
Eastern- Atlantic	1
Eastern- Central	1.15 (0.90-1.47)
Western- Prairies	**1.81 (1.38-2.38)**
Western- British Columbia	**1.94 (1.42-2.64)**
Northern territories	**3.02 (2.21-4.12)**
Mother's education level (years)	**1.08 (1.05-1.12)**
Age at first pregnancy (years)	**1.05 (1.03-1.07)**
Number of past pregnancies	**1.16 (1.09-1.23)**
BMI before pregnancy (Kg/m^2^)	**0.97 (0.95-0.99)**

† 95% CI were calculated using bootstrapping technique

‡ Eastern Atlantic: Newfoundland & Labrador, Nova Scotia, Prince Edward Island & New Brunswick; Eastern Central: Quebec & Ontario; Western Prairies: Manitoba, Saskatchewan, & Alberta; Western British Columbia: British Columbia; and Northern Territories: Yukon Territory, Nunavut & Northwest Territories.

## Discussion

The present study aimed to investigate the prevalence and predictors of exclusive breastfeeding at 6 months among mothers throughout the Canadian provinces and territories. Although ever breastfeeding was 90.3%, half of the Canadian mothers exclusively breastfed their babies for 3 months and only 13.8% of the mothers remained exclusively breastfeeding for 6 months.

The exclusive breastfeeding rates decreased considerably from 1 month to 6 months among Canadian mothers. In Norway the 1-month and 4-month exclusive breastfeeding rates were 90% and 44% as compared to 63.3% and 41.4% in the present study [26]. In Quebec (1999/2000), the exclusive breastfeeding rates were 62% and 35% for 1 and 4 months of infant's age [13]. The Canadian 6-month exclusive breastfeeding rate is comparable with other developed countries. In the United States, the prevalence of exclusive breastfeeding at 6 months was 11.3% [27], whereas it was 10.1% in Sweden [28] and 7% in Norway [26]. The study rate, however, is lower than the rate (17%) reported earlier in Canada in 2003 by Millar & Maclean (2005). Similarly, the provincial rates reported in the present study are lower than the 2003 Canadian study [14]. The 2003 prevalence rates, for example, in British Columbia, Alberta and Ontario, were reported to be 28%, 22% and 18% respectively, while, in the present study, they were been measured as 19.2%, 15.3% and 14.5% respectively. Only Quebec illustrated a fixed rate of 10% in both surveys while New Brunswick reported an increase from 8% in 2003 to 10.9% in our study. While no data is available in 2003 for the Northern territories, the prevalence of exclusive breastfeeding in this study was the highest (21.2%) as compared to all other provinces. The differences between the two studies might either be attributed to variations in study designs, sample selection and variable definitions or to an actual decline in the rate of exclusive breastfeeding in Canada.

At the multivariate analysis, years of education was the only significant socio-economic predictor of 6-month exclusive breastfeeding. The results are in accordance with the international [26,29] and Canadian literature [9,11,14]. Nationally, Millar and Maclean (2005) revealed that postsecondary education was positively associated with exclusive breastfeeding for the first 6 months of life. Similarly in Quebec, having a university diploma increased the odds of 4-month exclusive breastfeeding [11] and not completing high school was a risk factor for early breastfeeding termination in Ontario [9]. A higher level of maternal education seems to allow mothers to formulate well-informed decisions regarding the feeding practices used for their infant.

With regard to maternal characteristics, living with a partner, having had previous pregnancies, older age at pregnancy and lower pre-pregnancy BMI was found to be significantly associated with 6-month exclusive breastfeeding. The presence of a partner is likely to provide increased support for the mother, which may ease the feeding process and the choice to exclusively breastfeed for 6 months. Although studies regarding the association between marital status and breastfeeding are inconsistent [26], the result of the present study is in agreement with studies from Norway and Germany [26,29]. Previous Canadian studies, however, failed to demonstrate this association [11,14]. High parity was also found to be positively associated with 6-month exclusive breastfeeding. A dose response relationship between parity and breastfeeding has been previously documented in the literature [26,30]. Multipara mothers are suggested to have increased knowledge and self confidence from earlier breastfeeding experiences. By the same token, young age at first pregnancy decreased the likelihood of 6-month exclusive breastfeeding. Evidence in the literature provide consistent results of a positive association between breastfeeding duration and maternal age [11,26,29,31]. Study results are also in agreement with the literature whereby maternal pre-pregnancy BMI was found to be negatively associated with breastfeeding [32-35]. It has been postulated that heavy weight might interfere with prolactin production [32]. The psychological factors associated with heavy weight may also have an impact on breastfeeding initiation and duration [33].

Evidence of the present study suggests that smoking during pregnancy decreases the likelihood of 6-month exclusive breastfeeding. Lande et al. (2003) also reported the association between exclusive breastfeeding at 4 months and maternal smoking status after delivery to be OR = 0.40 (95% CI: 0.32, 0.50). In Canada, Albertan mothers who smoked during pregnancy were less likely to continue breastfeeding for longer periods [10]. In Southwestern Ontario, the presence of a smoker at home after delivery increased the risk of early breastfeeding termination [12].

In the present study, the place of delivery was associated with the 6 month duration of exclusive breastfeeding. Mothers giving birth at home were 5 times more likely to exclusively breastfeed than mothers giving birth at hospitals. This relationship can be attributed to the negative influence of formula supplementation in the hospital [9]. A study in a Canadian university teaching hospital reported that 47.9% of the infants received formula milk during hospital stay [36]. It is noteworthy, as well, that the characteristics of women giving birth at home are substantially different from their counterparts [37,38] which might reflect on their breastfeeding choices. Besides the place of delivery, the type of delivery was also related to exclusive breastfeeding status. Vaginal deliveries increased the odds of exclusive breastfeeding at 6 months. Pain and discomfort associated with Caesarean section may prevent the mother from breastfeeding. Results from the literature, however, are in disagreement about the relationship between the type of delivery and breastfeeding duration [30,39-41].

Infant's admission to intensive care unit and employment before 6 months from birth were negatively associated with exclusive breastfeeding. It has been reported by Jakobsen et al. (1996) that child illness is a common risk factor for shorter duration of breastfeeding. The impact of working shortly after delivery on breastfeeding termination has also been documented in previous studies [30,39]. The proximity of the nonworking mother to her child makes breastfeeding more accessible during the first 6 months of life.

The response rate in the present study was 75.2%. The main reason for non-response was the inability to establish contact with the mothers who were initially selected from the Canadian Census of Population. However, the population weights created by Statistics Canada and used in the analysis accounted for this non-response. The cross-sectional nature of the study and inability to measure the duration of breastfeeding longitudinally stands out as another limitation. The study would have also greatly benefited from information on the mother's knowledge and opinion on exclusive breastfeeding. Information on the support available to the mother during the prenatal and postnatal period would have been very helpful as well. Despite the above facts, this is the first nationwide study that assessed a comprehensive list of potential predictors for 6-month exclusive breastfeeding across the Canadian provinces and territories. The data is representative of the Canadian mother population. The confounding effect of many covariates is also well contained. Moreover, the recall bias of the outcome variables maybe reduced as mothers were surveyed within a year of the birth of their child.

## Conclusions

In Canada, almost half of the women are exclusively breastfeeding at 3 months while only 13.8% remain doing so at 6 months. Results of the present study constitute the basis for designing interventions targeting policy makers and health professionals in order to bridge the gap between the current practices of breastfeeding and the WHO recommendation. Single, less educated and nulliparous mothers should constitute a focus of these intervention programs. Finally, promoting exclusive breastfeeding rates for the first months of life is highly warranted.

## Abbreviations

BMI: Body mass index; CI: Confidence interval; MES: Maternity Experience Survey; OR: Odds Ratio; SAS: Statistical Analysis Software; SD: Standard deviation; SPSS: Statistical Package for Social Sciences; WHO: World Health Organization.

## Competing interests

The authors declare that they have no competing interests.

## Authors' contributions

BAS performed the analysis and the write up of the manuscript. AL assisted in the analysis and write up of the manuscript. MF provided technical support and advice on breastfeeding and reviewed the article. HT generated the idea of the research and supervised the analysis and write up of the manuscript.

All authors read and approved the final manuscript.

## Pre-publication history

The pre-publication history for this paper can be accessed here:

http://www.biomedcentral.com/1471-2431/10/20/prepub
